# Posterior Reversible Encephalopathy Syndrome (PRES) and intraventricular hemorrhage in a patient with untreated hypertension: a case report

**DOI:** 10.1186/s12245-026-01259-1

**Published:** 2026-05-28

**Authors:** Turamyimana Faustin, Sultan Menbeu Mohammad, Arlene Ndayisenga, Dagnachew Hilina

**Affiliations:** 1 Department of Emergency Medicine and Critical Care, University of Rwanda, Kigali, Rwanda; 2 African Health Science University, Kigali, Rwanda; 3 King Faisal Hospital, Rwanda, Kigali, Rwanda

**Keywords:** Posterior reversible encephalopathy syndrome, Hypertensive emergency, Intraventricular hemorrhage, Neurocritical care, Multidisciplinary management

## Abstract

**Background:**

Posterior Reversible Encephalopathy Syndrome (PRES) is a neurotoxic condition characterized by headache, altered mental status, seizures, and visual disturbances, frequently associated with vasogenic edema on neuroimaging. Although acute hypertension is a common precipitant, the occurrence of PRES with intraventricular hemorrhage is rare. This report documents this unusual combination, emphasizing its clinical significance. The limited prevalence of this presentation in published literature further underscores the importance of this case.

**Case presentation:**

A 39-year-old male with a one-year history of untreated hypertension presented with severe headache, agitation, and decreased consciousness. On admission, the patient presented with acute encephalopathy characterized by altered mental status, with a Glasgow Coma Scale (GCS) score of 13/15, and focal neurological deficits including left-sided hemiparesis. He had a known history of untreated hypertension and chronic heavy alcohol consumption, with no prior use of antihypertensive medications. There was no history of recent head trauma, fever, or illicit drug use. Initial vital signs revealed markedly elevated blood pressure consistent with a hypertensive emergency. Initial computed tomography (CT) of the brain showed extensive left lateral ventricular intraventricular hemorrhage (IVH) with hydrocephalus, necessitating emergency external ventricular drain (EVD) placement. Magnetic resonance imaging (MRI) confirmed PRES in the parieto-occipital regions. Aggressive antihypertensive therapy, supportive care, and multidisciplinary management led to significant neurological improvement.

**Conclusion:**

This case illustrates a severe presentation of PRES, with coexistence of intraventricular hemorrhage. Early recognition of hypertensive emergencies, careful management of complications, and timely, multidisciplinary interventions are essential for favorable neurological outcomes.

## Introduction

Posterior Reversible Encephalopathy Syndrome (PRES) is a clinico-radiological diagnosis first described by Hinchey et al., in 1996. Hinchey, [12] The classic presentation includes headache, encephalopathy, seizures, and visual disturbances.Fischer and Schmutzhard, [4], Hinduja, [13] Radiologically, it typically shows vasogenic edema in the posterior cerebral white matter. Hinduja, [13]However, atypical distributions are common and well recognized. McKinney, [16] The primary mechanisms are believed to be impaired cerebral autoregulation and endothelial dysfunction. Fugate and Rabinstein, [6]These lead to hyperperfusion, vasogenic edema, and capillary leakage Fugate and Rabinstein, [6].

Common precipitants include severe hypertension, eclampsia, renal disease, and immunosuppressive therapy.Fischer and Schmutzhard, [4] While often reversible, complications such as intracerebral hemorrhage (ICH) or ischemia can worsen the prognosis. McKinney, [16] We present a complex case of a young male with untreated hypertension. He developed PRES with intraventricular hemorrhage, illustrating a management challenge and the importance of a multidisciplinary approach.

## Case presentation

### Patient information & history

A 39-year-old male with a known history of hypertension diagnosed one year prior, presented with a one-day history of severe, thunderclap headache followed by disorientation and agitation. He had been non-adherent to any antihypertensive medication. He had a known history of untreated hypertension and chronic heavy alcohol consumption, with no prior use of antihypertensive medications. There was no history of recent head trauma, fever, or illicit drug use, autoimmune diseases, immunosuppressant medications, or chemotherapy. No HIV nor other infectious diseases.

On admission, Initial vital signs revealed markedly elevated blood pressure (200/110 mmHg) consistent with a hypertensive emergency. The patient presented with acute encephalopathy characterized by altered mental status, with a Glasgow Coma Scale (GCS) score of 13/15 (E4M5V4), and focal neurological deficits, including left-sided hemiparesis and no meningeal signs recognized. The other physical examination was unremarkable.

### Diagnostic investigations

#### Initial CT Brain on day one

**Fig. 1:**
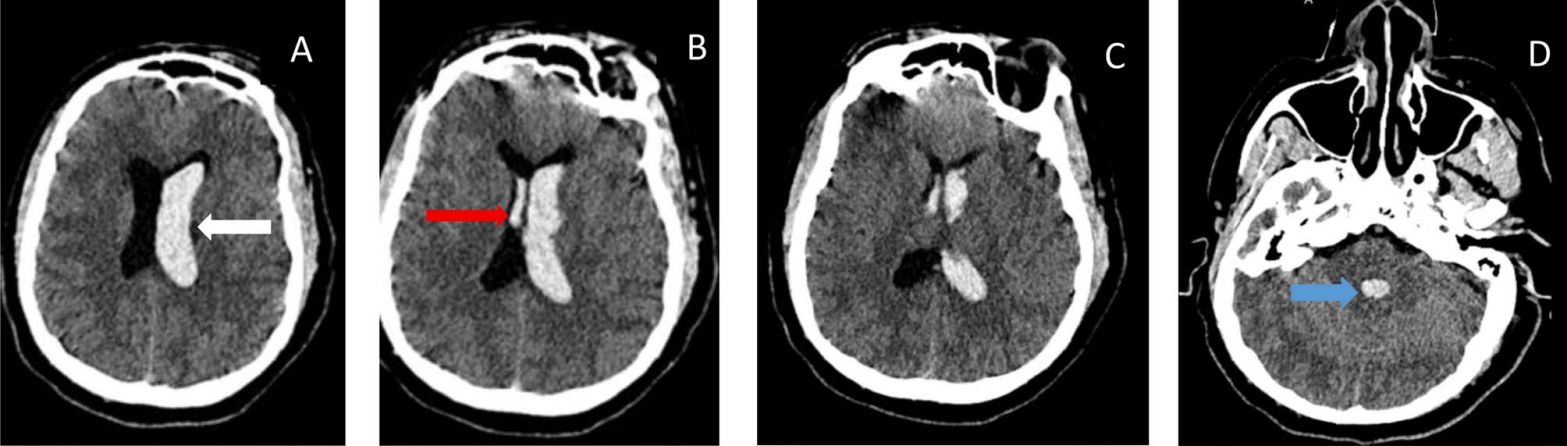
Initial non-contrast axial CT scan showing Images demonstrate extensive acute intraventricular hemorrhage (IVH). Hyperdense blood fills the left lateral ventricle (white arrow, image **A**), third ventricle (red arrow, Image **B**), and fourth ventricle (blue arrow, image **D**), indicating hemorrhage primarily confined to the ventricular system. There is no evidence of a large parenchymal hematoma or significant mass effect. The findings are consistent with hypertensive hemorrhage, likely secondary to the severe hypertension precipitating Posterior Reversible Encephalopathy Syndrome (PRES)

Revealed extensive acute intraventricular hemorrhage (IVH) throughout the ventricular system with associated hydrocephalus (Fig. 1). Unfortunately, CT angiography was not done due to kidney injury. MRI was done 15days later, after a CT scan control showed new ischemic lesions.

**Fig. 2:**
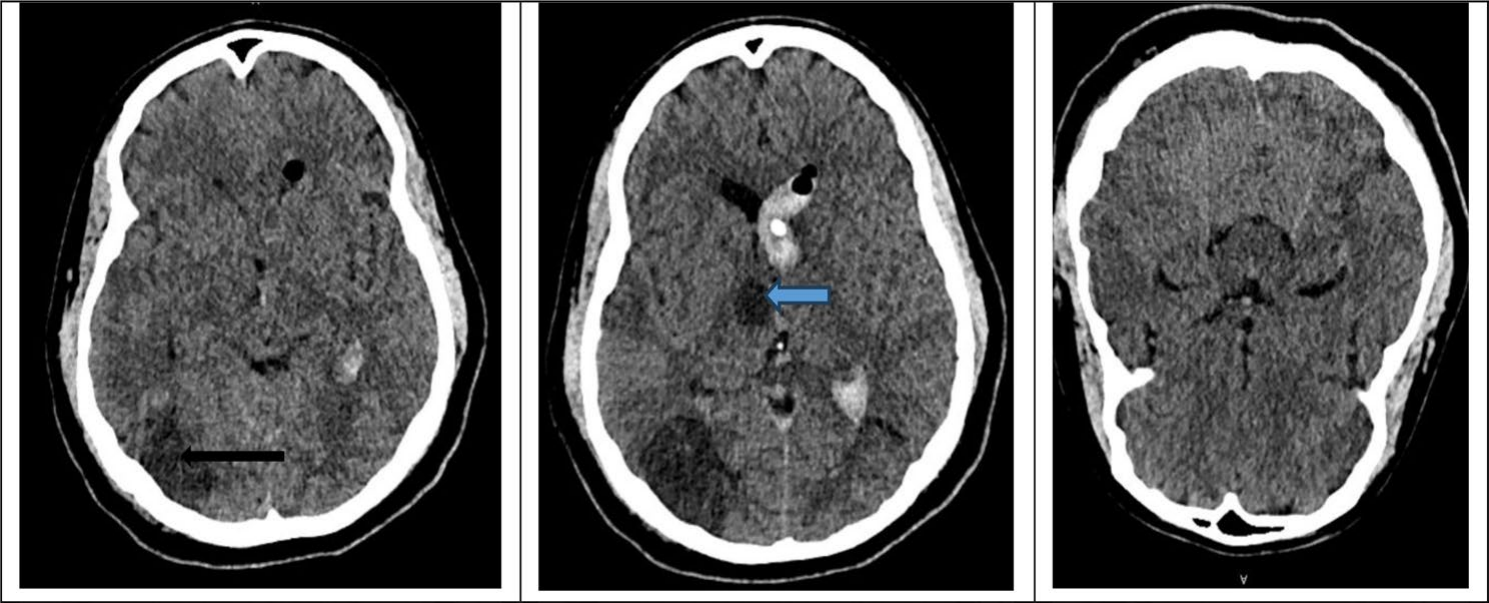
CT brain control done 5 days after 1st CT scan, showing new ischemic lesion in the left occipital region (black arrow) and in the left thalamus (blue arrow)

Brain MRI was performed after follow-up CT imaging, obtained five days after ICU admission, demonstrated new ischemic-appearing lesions in the left occipital lobe and left thalamus (Fig. 2). MRI acquisition was initially deferred due to the patient’s clinical instability and the prolonged imaging time; it was subsequently performed 1 week later, once the patient was sufficiently stable for safe transport and completion of the examination.

**Fig. 3:**
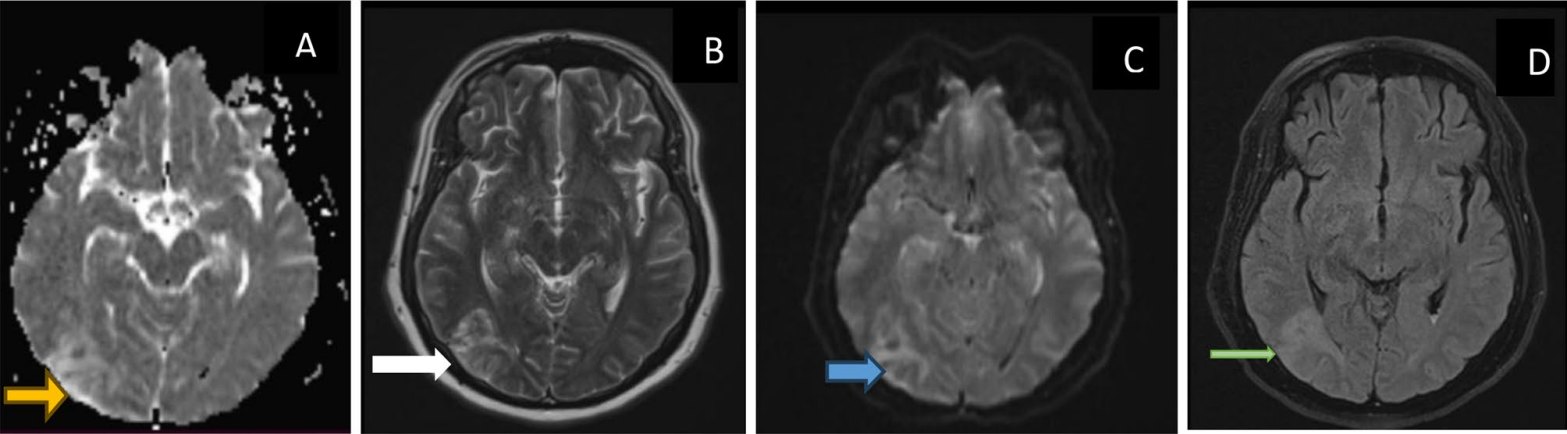
MRI Brain demonstrating sequences in axial views, including axial T2, FLAIR, DWI/ADC, and GRE/SWI. (**A**) Apparent Diffusion Coefficient (ADC) map shows increased signal (yellow arrow) predominantly affecting cortical and subcortical white matter of the left occipital lobe, consistent with vasogenic edema. (**B**) Axial T2-weighted Turbo Spin Echo (TSE) demonstrates corresponding areas of high T2 signal intensity (white arrow) within the cortical and subcortical white matter of the left occipital lobe. (**C**) Diffusion-weighted trace image (b = 0) reveals confluent regions of increased signal intensity (blue arrow) in the occipital cortical and subcortical white matter, supporting vasogenic rather than cytotoxic edema. (**D**): Axial T2-weighted FLAIR image shows extensive areas of hyperintensity (green arrow) predominantly involving the occipital & parietal cortical and subcortical white matter. This posterior-predominant distribution is characteristic of Posterior Reversible Encephalopathy Syndrome (PRES). These images, along with severe uncontrolled hypertension and acute encephalopathy, these findings are consistent with PRES with intraventricular hemorrhagic complications

Brain MRI revealed extensive vasogenic edema predominantly affecting the posterior regions of the left cerebral hemisphere (Fig. 3). Apparent diffusion coefficient maps demonstrated increased signal involving the cortical and subcortical white matter of the left occipital and parietal lobes, with corresponding hyperintensity observed on T2-weighted and FLAIR sequences. Diffusion-weighted imaging showed increased signal without evidence of diffusion restriction, which is consistent with vasogenic rather than cytotoxic edema. The posterior-predominant distribution of cortical and subcortical involvement is characteristic of posterior reversible encephalopathy syndrome (PRES). In the setting of severe uncontrolled hypertension and acute encephalopathy, these MRI findings are indicative of PRES with associated intraventricular hemorrhagic complications.

#### Laboratory Studies

Significant findings included elevated creatinine (159 µmol/L), suggesting hypertensive nephropathy. The renal artery Doppler ultrasound was normal; the endocrine workup, including electrolytes, was unremarkable; and HIV was negative. Coagulation studies (PT, aPTT, INR) were within normal limits.

#### Echocardiography

Showed moderate left ventricular hypertrophy (LVH) with grade I diastolic dysfunction, consistent with chronic hypertension.

### Management & hospital course

The patient’s management was complex and protracted:

#### Neurosurgical Intervention

An initial brain CT scan was immediately done, showing IVH but without features of ischemic stroke or localized vasogenic brain edema. Due to obstructive hydrocephalus from IVH, an emergency EVD was inserted in the left lateral ventricle on admission day. The EVD remained in place for seven days, draining bloody cerebrospinal fluid, and was removed once the hydrocephalus resolved.

#### Hypertension Management

Initially, the blood pressure was poorly controlled by intravenous infusion of labetalol and other oral antihypertensive medications to lower the high blood pressure, until later rapidly, the blood pressure was controlled by oral medicines, including nifedipine, captopril, and metoprolol, but eventually, a combination of Carvedilol and Exforge HCT (Amlodipine/Valsartan/Hydrochlorothiazide) were utilized to achieve sustainable control. Blood pressure targets were initially set at a systolic pressure of 140–160 mmHg to ensure adequate cerebral perfusion.

#### ICU & Supportive Care

The patient was managed in the ICU, intubated, as he was not able to protect his airway, with close neurological monitoring, seizure prophylaxis with Phenytoin, analgesia, and management of episodes of fever (since day 7 of EVD insertion), empirically treated as hospital-acquired infections (treated with vancomycin) despite that septic workups done were negative. The patient’s neurological status improved, allowing extubation, with Glasgow Coma Scale (GCS) returning to 15/15, and he was transferred to the general ward for continuation of rehabilitation.

**Fig. 4:**
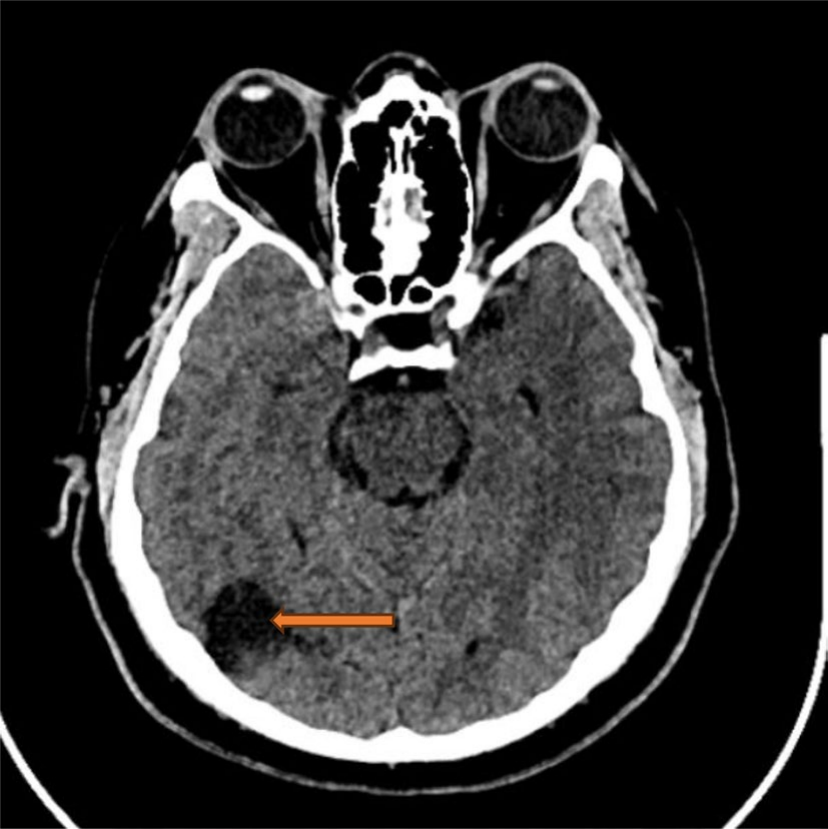
Follow-up non-contrast axial brain CT scan (4 months post-PRES). The image demonstrates chronic sequelae in the left occipital region (brown arrow), showing low attenuation consistent with encephalomalacia resulting from prior posterior reversible encephalopathy syndrome. The previously seen intraventricular hemorrhage has resolved, with the ventricular system now filled with cerebrospinal fluid. In this case, PRES was not reversible, contrary to its traditional characterization, highlighting that the syndrome is not invariably reversible

At discharge, blood pressure was adequately controlled with a multi-drug antihypertensive regimen, and multidisciplinary follow-up was arranged. At two months post-discharge, the patient demonstrated continued neurological improvement, with only mild residual left-sided weakness. By four months, cognitive function had fully recovered, and the patient had resumed all pre-illness activities. Cognitive assessment was performed via structured telephone follow-up using a focused clinical interview assessing orientation, memory, attention, language, and executive functioning, which revealed no residual cognitive deficits. Blood pressure remained well controlled. A follow-up CT scan performed four months after discharge demonstrated complete resorption of the intraventricular hemorrhage, with evolution of the previously affected PRES regions into areas of encephalomalacia (Fig. 4).

## Discussion

This case represents a severe and atypical manifestation of posterior reversible encephalopathy syndrome (PRES) complicated by massive intraventricular hemorrhage (IVH). Long-standing, untreated hypertension likely precipitated a catastrophic failure of cerebral autoregulation, resulting in endothelial dysfunction and vasogenic edema characteristic of PRES [4]. Whether extreme blood pressure elevations directly caused vascular rupture with subsequent IVH, or whether hemorrhage developed secondary to blood–brain barrier disruption intrinsic to PRES, remains uncertain [18].

Although PRES is traditionally considered non-hemorrhagic, intracranial hemorrhage occurs in approximately 15–20% of cases, most commonly as intracerebral or subarachnoid hemorrhage. Isolated or predominant IVH is distinctly rare [1, 2, 16, 20]. The coexistence of PRES and IVH represents a particularly severe phenotype, frequently associated with hydrocephalus, intracranial hypertension, prolonged ICU stay, and worse neurological outcomes [14]. Nevertheless, several case reports, including an adolescent with primary IVH complicating PRES, demonstrate that meaningful neurological recovery is possible with aggressive supportive care and intracranial pressure management [10, 17].

MRI was pivotal in establishing the diagnosis by demonstrating characteristic parieto-occipital vasogenic edema despite extensive IVH. MRI remains the imaging modality of choice for PRES, particularly in atypical or hemorrhagic presentations, where CT findings may be misleading [6, 16].

Blood pressure management posed a major therapeutic challenge. Controlled reduction was essential to limit hyperperfusion, prevent progression of vasogenic edema, and reduce the risk of IVH expansion [4, 6, 11]. Conversely, excessive lowering risked cerebral hypoperfusion in regions with impaired autoregulation, potentially converting reversible edema into ischemic injury [9]. In line with contemporary hemorrhagic stroke guidance, a cautious systolic blood pressure target of approximately 140 mmHg was pursued using titratable intravenous agents, acknowledging that optimal targets in combined PRES and IVH remain undefined and require individualization [11, 15].

The severity of IVH necessitated external ventricular drainage to manage hydrocephalus and intracranial hypertension. While life-saving, EVD placement carries a significant infection risk, which complicated this patient’s course [3]. Despite this, subsequent imaging evolution supported PRES as the primary pathological process, with IVH representing a severe secondary complication rather than the initiating event [17].

Beyond hypertension, intraventricular hemorrhage (IVH) has a broad differential diagnosis that must be considered, particularly in atypical or severe presentations. Vascular lesions such as arteriovenous malformations, cavernous malformations, and ruptured intracranial aneurysms are well-recognized causes of primary or predominant IVH, especially in younger patients or in the absence of chronic hypertension [7, 8]. Coagulopathies, including anticoagulant use, thrombocytopenia, liver disease, and hematologic disorders, significantly increase the risk of spontaneous IVH [5]. Intracranial tumors, particularly intraventricular or hemorrhage-prone neoplasms, may also present with IVH due to tumor-related vascular fragility [21]. Traumatic brain injury remains another important cause, even following minor or unwitnessed trauma [8]. In the present case, the absence of vascular malformations or aneurysms on imaging, lack of traumatic history, and no evidence of coagulopathy or neoplasm support hypertension-related vascular failure in the setting of PRES as the most plausible mechanism.

The patient’s favourable neurological recovery, with only mild residual hemiparesis, aligns with prior reports and underscores that good outcomes are achievable even in hemorrhagic and intraventricular forms of PRES when timely blood pressure control, intracranial pressure management, and multidisciplinary neurocritical care are provided [19].

This report is limited by its single-case design, precluding causal inference. Additionally, chronic alcohol use represents a potential confounder, given its association with hypertension and endothelial dysfunction. Larger case series are needed to elucidate mechanisms better, define optimal blood pressure targets, and refine prognostic stratification in this rare yet high-risk PRES phenotype. The interpretation of neuroimaging findings was limited by the absence of a dedicated neuroradiology specialist at our institution, which may have constrained the depth of radiological analysis and image annotation in this case.

## Conclusion

This case shows that posterior reversible encephalopathy syndrome (PRES) is a critical diagnosis to consider in patients presenting with acute encephalopathy and severe hypertension, even with concurrent intracranial hemorrhage. Accurate diagnosis and characterization of PRES, particularly in atypical or hemorrhagic presentations, benefits significantly from expert neuroradiological input. Optimal patient outcome depends on prompt recognition via MRI, meticulous acute blood pressure control managed by a multidisciplinary team, and robust long-term strategies to ensure adherence to antihypertensive therapy, preventing recurrence.

## Data Availability

No datasets were generated or analysed during the current study.
